# Multiple and diverse structural changes affect the breakpoint regions of polymorphic inversions across the Drosophila genus

**DOI:** 10.1038/srep36248

**Published:** 2016-10-26

**Authors:** Eva Puerma, Dorcas J. Orengo, Montserrat Aguadé

**Affiliations:** 1 Departament de Genètica, Microbiologia i Estadística, Facultat de Biologia and Institut de Recerca de la Biodiversitat (IRBio), Universitat de Barcelona, Barcelona, Spain

## Abstract

Chromosomal polymorphism is widespread in the Drosophila genus, with extensive evidence supporting its adaptive character in diverse species. Moreover, inversions are the major contributors to the genus chromosomal evolution. The molecular characterization of a reduced number of polymorphic inversion breakpoints in *Drosophila melanogaster* and *Drosophila subobscura* supports that their inversions would have mostly originated through a mechanism that generates duplications —staggered double-strand breaks— and has thus the potential to contribute to their adaptive character. There is also evidence for inversion breakpoint reuse at different time scales. Here, we have characterized the breakpoints of two inversions of *D. subobscura —*O_4_ and O_8_*—* involved in complex arrangements that are frequent in the warm parts of the species distribution area. The duplications detected at their breakpoints are consistent with their origin through the staggered-break mechanism, which further supports it as the prevalent mechanism in *D. subobscura*. The comparative analysis of inversions breakpoint regions across the Drosophila genus has revealed several genes affected by multiple disruptions due not only to inversions but also to single-gene transpositions and duplications.

Chromosomal inversions were first identified in *Drosophila melanogaster* through their recombination suppressing effect in heterozygotes^1^. The presence of polytene chromosomes in insects facilitated their subsequent cytological identification in this and other Drosophila species, which opened up the possibility to detect and identify inversions that segregated in natural populations. Dobzhansky pioneered the study of chromosomal polymorphism in natural populations by performing extensive geographical and temporal surveys of inversion frequencies in the Nearctic species *D. pseudoobscura* (as compiled in Lewontin *et al*.^2^). This work prompted similar studies in other Drosophila species such as *D. subobscura*, *D. repleta* and *D. melanogaster* (as summarized in Krimbas and Powell^3^). These studies provided ample evidence for the adaptive character of chromosomal polymorphism.

In the Drosophila genus, detailed cytological maps based on the banding pattern of polytene chromosomes were built for many species. The availability of these maps allowed locating inversion breakpoints, and it later allowed combining the use of molecular markers and *in situ* hybridization to molecularly identify and characterize these breakpoints. This is however a laborious endeavor that in the absence of a medium to high quality reference genome sequence of the species under study requires the availability of either other genomic resources such as BAC libraries to narrow down the region under scrutiny, or a good reference genome of a relatively closely related species to use as a guide. The number of polymorphic inversions with breakpoints molecularly characterized is still scanty for Drosophila species^4^,^5^,^6^,^7^,^8^,^9^,^10^,^11^,^12^,^13^,^14^. Only in *D. melanogaster* with one of the best reference genomes, the availability of population genome-wide datasets has allowed to bioinformatically identify the breakpoints of nine polymorphic inversions^15^,^16^,^17^,^18^,^19^.

Classical cytological studies of inversion polymorphism in different Drosophila species had revealed that inversion breakpoints are not evenly distributed either among or along chromosomal arms^20^,^21^. Moreover, they provided evidence for breakpoint reuse at this short time scale. The comparative analysis of genome sequences across the Drosophila phylogeny also revealed the uneven distribution of the breakpoints of fixed chromosomal rearrangements, providing evidence that some regions had been multiply disrupted^22^,^23^. The observed reuse of some breakpoints or breakpoint regions can reflect the breakage-prone character (or fragility) of some genomic regions. However, evolutionary successful rearrangements (both polymorphic and fixed) constitute a subset of those generated. The observed reuse can therefore also reflect the new arrangement effect on fitness.

The characterization of inversion breakpoints in inverted and non-inverted chromosomes allows addressing various important questions concerning among others the possible functional effect of the inversion itself, and the repeated use of breakpoint regions. Inversions can originate through (i) the cut-and-paste mechanism, (ii) unequal recombination between repetitive elements, and (iii) staggered double-strand breaks and their subsequent repair. However, only the latter mechanism generates segmental duplications at the breakpoints of the inverted arrangement^24^. The characterization of inversion breakpoints also facilitates to later uncover putative targets of selection in the inverted fragment through its effect on nucleotide variation. Our work focusing on five inversions of the E chromosome (Muller’s C element) of *D. subobscura* that are involved in chromosomal arrangements of the E_1+2_ complex, has already unveiled that one inversion breakpoint with cytological evidence for having been multiply reused has also been multiply reused molecularly, and that a second breakpoint also considered to be cytologically shared by two inversions is not coincident at the molecular level^12^,^14^. Moreover, the molecular structure of these inversions breakpoints and of those of one inversion of the O chromosome (Muller’s E element)^11^ has also revealed that the staggered-breaks mechanism is probably the most frequently used mechanism to generate inversions in this species. Finally, the comparative analysis of the breakpoint regions of the *D. subobscura* polymorphic inversions across the Drosophila genus has revealed that some of these regions have been multiply disrupted^12^,^13^,^14^.

Here we have characterized the breakpoints of two additional inversions of the O chromosome of this species —inversions O_4_ and O_8_— that, like the five inversions of the E chromosome with breakpoints previously characterized, are involved in generating complex arrangements. Indeed, inversions O_4_ and O_8_ are overlapping inversions that occurred sequentially and led from the ancestral and now extinct O_3_ arrangement to the extant O_3+4_ and O_3+4+8_ arrangements (Fig. 1). These arrangements can be present in natural populations also harboring O_st_ that, like O_3+4_, originated from the ancestral arrangement, in this case through the O_3_ inversion (Fig. 1). The frequencies of O_3+4_ and O_3+4+8_ in the species ancestral distribution area do not only exhibit a high negative correlation with latitude^25^ but also a high positive correlation with temperature^26^, which has led them to be considered warm-adapted arrangements^27^. The identification and molecular characterization of inversions O_4_ and O_8_ breakpoint regions of *D. subobscura* will further contribute to our understanding of how polymorphic inversions originate and it will also pave the road to identify the genetic basis of their adaptive character. Additionally, the characterization of these regions across the Drosophila genus will shed light on the extent of breakpoint reuse at different time scales.

## Results

### Identification of breakpoint regions by chromosome walking

We have identified the breakpoints of two inversions —inversions O_4_ and O_8_ of the O chromosome— by performing the corresponding chromosomal walks that were guided by the results obtained by *in situ* hybridization of the different rounds of probes in both non-inverted and inverted chromosomes. Each walk was initiated from a molecular marker that had been previously mapped near the corresponding breakpoint.

#### O_4_ inversion breakpoints

**Figure 1:**
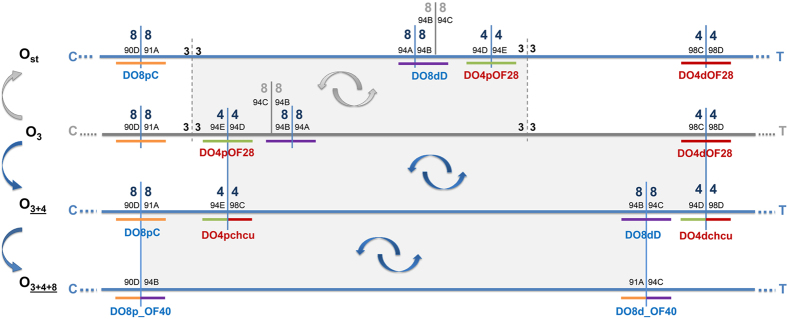
Schematic representation of the O chromosome regions of *Drosophila subobscura* affected by inversions O_3_, O_4_ and O_8_. The affected regions are represented in extant gene arrangements O_st_, O_3+4_ and O_3+4+8_, and in the extinct ancestral O_3_ chromosomal arrangement of *Drosophila subobscura*. Horizontal lines represent the different chromosomal arrangements (blue if extant and grey if extinct). Short vertical lines on the O_st_ arrangement represent the different inversion breakpoints with indication of their location (section) on the Kunze-Mühl and Müller^28^ map. Double arrows highlight inversion events between arrangements whereas arrows on the left side of the image represent the sequential accumulation of inversions from the ancestral O_3_ arrangement (grey, the previously characterized inversion O_3_; blue, inversions O_4_ and O_8_). Grey shaded boxes between arrangements indicate the extent of the corresponding inversion. Short colored horizontal lines represent the fragments spanning the breakpoint regions of the inversions. C, centromere; T, telomere.

The breakpoints of inversion O_4_ are cytologically located at sections 94D/94E and 98C/98D on the O_st_ Kunze-Mühl and Müller^28^ map and therefore between sections 94E/94D and 98C/98D on the ancestral O_3_ arrangement (Fig. 1). Markers *AbdA*^11^ and *Obp83a/Obp83b*^29^ that are located at section 94E and 98D, respectively, were used as starting points to identify the inversion breakpoints in non-inverted chromosomes.

For the proximal breakpoint, three rounds of serial *in situ* hybridizations were needed to design a final probe that putatively would span it (Supplementary text and Supplementary Figure S1). This probe —DO4pOF28— gave a single strong signal at section 94D/94E when hybridized on O_st_ chromosomes, and two strong signals at sections 94E next to 98C and 94D next to 98D when hybridized on O_3+4_ chromosomes (Supplementary Figure S2). These results confirmed that this probe spanned the proximal breakpoint of inversion O_4_ in non-inverted (O_st_ and O_3_) chromosomes.

For the distal breakpoint, two rounds of serial *in situ* hybridizations were needed to design a final probe that would putatively span it (Supplementary text and Supplementary Figure S1). This probe —DO4dOF28—gave a single strong signal at section 98C/98D on O_st_ chromosomes, and two strong signals at sections 98C next to 94E and 98D next to 94D on O_3+4_ chromosomes (Supplementary Figure S2). These results confirmed that this probe spanned the distal breakpoint of inversion O_4_ in non-inverted (O_st_ and O_3_) chromosomes.

The identification of both the proximal and distal breakpoints of inversion O_4_ in O_st_ chromosomes allowed amplifying the fragments spanning these breakpoints in O_3+4_ chromosomes with the corresponding combination of oligonucleotides (Fig. 1). Their *in situ* hybridization on O_st_ chromosomes gave two signals (Supplementary Figure S2), which confirmed that they included the corresponding breakpoints in O_3+4_ chromosomes. It should be added that they also gave two signals on O_3+4_ chromosomes (see next paragraph).

**Figure 2:**
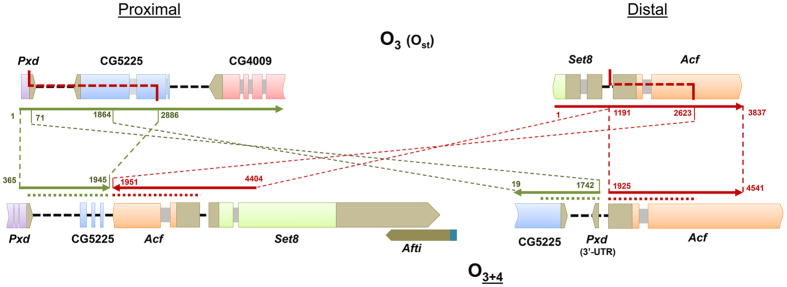
Schematic representation of inversion O_4_ breakpoint regions in chromosomal arrangements O_3_ and O_3+4_. Colored arrows represent the breakpoint regions annotated as in Fig. 1. Colored and grey boxes represent gene exons and introns, respectively, whereas black dashed lines represent intergenic regions. Red dashed lines along a chromosomal region represent staggered breaks and their limits, whereas dashed lines between chromosomal arrangements indicate the limits and orientation of homologous regions, with numbers indicating their location in the sequenced fragments.

The fragments spanning the breakpoints in O_st_ and O_3+4_ chromosomes were completely sequenced and subsequently annotated. The ~5.6-kb long fragment spanning the proximal breakpoint in O_st_ —DO4pOF28— contains part of the *Pxd* gene, the CG5225 gene and part of the CG4009 gene, whereas the ~3.8-kb long fragment spanning the distal breakpoint —DO4dOF28— contains part of both the *Set8* and *Acf* genes (Fig. 2). In O_3+4_, the ~8.7-kb long fragment —DO4pchcu— spanning the proximal breakpoint contains part of the *Pxd* and *Acf* genes, and the *Set8* gene, whereas the ~5.6-kb long fragment spanning the distal breakpoint —DO4dchcu— contains part of the CG5225 and *Acf* genes (Fig. 2). The pairwise comparison of fragments spanning the breakpoints in O_st_ and O_3+4_ chromosomes allowed delimiting and characterizing the breakpoints. The presence in the O_3+4_ proximal breakpoint of part of the *Acf* gene and remnants of the CG5225 gene, and the presence in the distal breakpoint of part of the *Pxd* gene indicate that these fragments were duplicated during the inversion process. These results would therefore be solely consistent with the O_4_ inversion having originated through the staggered double-strand break mechanism (Fig. 2). The duplicated fragments would explain the double signal observed at the breakpoint regions in both the O_st_ and O_3+4_ arrangements when using as probes the O_3+4_ breakpoints.

#### O_8_ inversion breakpoints

The breakpoints of inversion O_8_ are cytologically located at sections 90D/91A and 94A/94B (Fig. 1) according to the Kunze-Mühl and Müller^28^ map. Markers previously located near each breakpoint —DP2_4d at section 91A and *trus* at section 93D— were used to initiate the corresponding chromosomal walks.

For the proximal breakpoint, four rounds of serial *in situ* hybridizations were needed to identify the probe that putatively spanned the breakpoint (Supplementary text and Supplementary Figure S3). The DO8pC probe gave a single signal at section 90D/91A on O_3+4_ (*ch cu*) chromosomes and two strong signals at the corresponding sections on O_3+4+8_ (OF40) chromosomes (Supplementary Figure S4). These results confirmed that this probe spanned the proximal breakpoint of inversion O_8_ in non-inverted (O_3+4_) chromosomes.

For the distal breakpoint, three rounds of serial *in situ* hybridizations were needed to design a final probe that putatively spanned it (Supplementary text and Supplementary Figure S3). This probe —DO8dD— gave a single strong signal at section 94B/C on O_3+4_ (*ch cu*) chromosomes and two strong signals at the corresponding sections on O_3+4+8_ (OF40) chromosomes (Supplementary Figure S4). This result confirmed that this probe spanned the distal breakpoint of inversion O_8_ in non-inverted (O_3+4_) chromosomes, and that this breakpoint is located at section 94B/C of the Kunze-Mühl and Müller^28^ map and not at section 94A/B as previously described^28^.

The identification of both the proximal and distal breakpoints of inversion O_8_ in O_3+4_ chromosomes allowed amplifying the fragments spanning these breakpoints in the O_3+4+8_ chromosomes with the corresponding combination of oligonucleotides (Fig. 1). Their *in situ* hybridization on O_3+4_ chromosomes gave two signals (Supplementary Figure S4), a confirmation that they included the corresponding breakpoints in O_3+4+8_ chromosomes. It should be noted that they also gave two signals on O_3+4+8_ chromosomes (see next paragraph).

**Figure 3:**
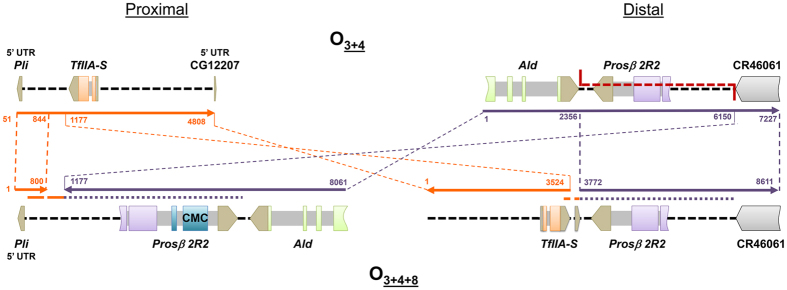
Schematic representation of inversion O_8_ breakpoint regions in chromosomal arrangements O_3+4_ and O_3+4+8_. Colored arrows represent the breakpoint regions annotated as in Fig. 1. Colored and grey boxes represent gene exons and introns, respectively, whereas black dashed lines represent intergenic regions. Red dashed lines along a chromosomal region represent staggered breaks and their limits, whereas dashed lines between chromosomal arrangements indicate the limits and orientation of homologous regions, with numbers indicating their location in the sequenced fragments.

The fragments spanning the breakpoints in O_3+4_ and O_3+4+8_ chromosomes were completely sequenced and subsequently annotated. The ~4.8-kb long fragment spanning the proximal breakpoint in O_3+4_ —DO8pC— contains genes *Pli* (partial), *TfIIA-S* and CG12207 (partial), whereas the ~7.2-kb long fragment spanning the distal breakpoint region —DO8dD— contains genes *Ald* (partial) and *Prosβ2R2*, and part of the ncRNA CR46041 gene (Fig. 3). In O_3+4+8_ (OF40) chromosomes, the ~8.0-kb long fragment spanning the proximal breakpoint —DO8p_OF40— contains genes *Pli* (partial), *Prosβ2R2* (with a CMC transposable element insertion) and *Ald* (partial), whereas the ~8.6-kb long fragment spanning the distal breakpoint —DO8d_OF40— contains genes *TfIIA-S* and *Prosβ2R2*, and part of the CR46041 gene (Fig. 3). The pairwise comparison of fragments spanning the breakpoints in O_3+4_ and O_3+4+8_ chromosomes allowed delimiting and characterizing the breakpoints. The proximal O_8_ inversion breakpoint in O_3+4_ arrangement can be narrowed down to an ~300-bp long stretch. Two small fragments (380- and 128-bp long) flanking this stretch are duplicated at the proximal and distal breakpoints of the O_3+4+8_ arrangement, respectively. The presence of the *Prosβ2R2* gene in the O_3+4+8_ proximal and distal breakpoints indicates that this gene was duplicated during the repair of the staggered double-strand break that initiated the inversion process.

### Inversions O_4_ and O_8_ breakpoint regions in Drosophila

A comparative analysis was performed across the Drosophila phylogeny relative to colinearity breaks near the genes either affected by or flanking the breakpoints of the here studied inversions as well as their immediate neighbors.

#### Breakpoint regions of the O_4_ inversion

The comparative analysis of the O_4_ proximal breakpoint region revealed that the four-genes block present in the O_st_ (O_3_) arrangement of *D. subobscura* —CG4009-CG5225-*Pxd*-CG8907*—* predated the diversification of the Drosophila genus given its presence in species of both the Drosophila and Sophophora subgenera (*e.g*., *D. mojavensis* and *D. subobscura* O_st_ arrangement, respectively; Supplementary Figure S5). However, the presence of gene CG31268 between genes CG4009 and CG5225 in most species of the Sophophora subgenus raises the possibility of a five-genes block — CG4009-CG31268-CG5225-*Pxd*-CG8907—predating the subgenera split. In the former scenario, gene CG31268 would have been inserted in the Sophophora subgenus ancestor whereas in the second scenario, this gene would have been lost in the ancestor of the Drosophila subgenus. In either case, gene CG31268 would have been subsequently either lost or transposed elsewhere in *D. subobscura*, and become a pseudogene in *D. simulans*. The CG5225 gene that would have been lost independently in *D. sechellia* and *D. grimshawi* would have undergone an intrachromosomal transposition in the ancestor of *D. pseudoobscura* and *D. persimilis* and a microinversion in the ancestor of the melanogaster group. Genes GA31730 in *D. pseudoobscura* and GL24546 in *D. persimilis* that exhibit fragments of similarity to gene CG5225 could be either a remnant of the latter gene duplicative transposition or the result of a new gene insertion. Concerning the two genes flanking the proximal O_4_ inversion breakpoint, a total of at least five intergenic disruptions would have occurred under the first scenario (four under the second scenario) across the Drosophila phylogeny —three (two) at the 5’ upstream region of gene CG5225 and two (two) at its 3’ downstream region— as a result of this gene intrachromosomal transposition and inversion, and the insertion of gene CG31268. Moreover, a disruption affecting genes *Pxd* and CG5225 would have originated the paracentric inversion that segregates in *D. subobscura* as part of the O_3+4_ arrangement.

The comparative analysis of the O_4_ distal breakpoint region —genes CG42233-*Acf-Set8-Afti*—revealed that this four-genes block is highly conserved across the Drosophila phylogeny. Indeed, only two disruptions would have occurred in the genus and both would have affected the same intergenic region (between genes *Set8* and *Acf*). Both disruptions can be considered the result of paracentric inversions: inversion O_4_ originated in *D. subobscura*, and at least a second inversion predating the melanogaster subgroup diversification.

#### Breakpoint regions of the O_8_ inversion

The comparative analysis of the O_8_ breakpoint regions revealed that they both have a rather complex evolutionary history (Supplementary Figure S6). For the proximal breakpoint that is flanked by genes *Pli* and *TfIIA-S*, the presence of the *Lsp1β-Pli-TfIIA-S* block in species of the Drosophila subgenus as well as in species of the obscura group, constitutes a clear indication of the ancestral character of this 3-genes block. In contrast, the repeated disruption of the upstream region of gene *TfIIA-S* by at least three paracentric inversions precludes inferring its ancestral neighbor. Moreover, at the downstream region of gene *Pli*, the *Lsp1β* gene would have been the subject of two independent duplications plus a microinversion and an interchromosomal transposition to Muller’s B element in the ancestor of the melanogaster subgroup, and it also flanked the breakpoint of a paracentric inversion. Concerning the two genes flanking the proximal O_8_ inversion breakpoint, a total of at least seven intergenic disruptions would have occurred across the Drosophila phylogeny —three at the 5′ upstream region of gene *TfIIA-S*, three at the 3′ downstream region of the *Pli* gene, and one between both genes— as a result of four paracentric inversions, a microinversion and a gene transposition.

The edges of the *Prosβ2R2* gene delimit the O_8_ distal breakpoint. The comparative analysis of this gene and its two neighbors in *D. subobscura* —*Ald*-*Prosβ2R2*-CR31086*—* revealed that this 3-genes block is only present in species of the obscura group. However, when an extended 5-genes block was considered —CG6154-*Ald*-*Prosβ2R2*-CR31086-CG12290—, we could detect that the four genes flanking *Prosβ2R2* were present as a block in *D. melanogaster* and likely also in the remaining nine species as revealed by the three protein coding genes. In the obscura group species, there is a second inverted copy of *Prosβ2R2* separated by three coding regions. Moreover, only in species of this group and the melanogaster subgroup is *Prosβ2R2* located in Muller’s E element. Its localization in Muller’s A element of *D. ananassae* and in Muller’s D element of species of the Drosophila subgenus indicates that this gene would have been the subject of at least one interchromosomal transposition. It would have been also affected by at least one intrachromosomal transposition predating the diversification of the obscura group (Supplementary Figure S6), as well as by the paracentric inversion that segregates in *D. subobscura* as part of the O_3+4+8_ arrangement and a one-gene duplication and its intrachromosomal transposition in the ancestor of the obscura group.

## Discussion

Chromosomal inversion polymorphism is widespread in the Drosophila genus even though it is unevenly distributed across species and also among chromosomal elements of polymorphic species. *Drosophila subobscura* stands out because its five large acrocentric chromosomes are polymorphic. Muller elements C and E of this species (chromosomes E and O, respectively) are those for which the highest numbers of naturally occurring inversions have been described^30^. Moreover, both elements present complex systems of inversions—the E_1+2_ and O_3+4_ complexes, respectively— resulting from the sequential accumulation of inversions. Upon completing the characterization of the breakpoints of the five inversions of the E chromosome leading from the ancestral E_st_ arrangement to the four most common arrangements of the E_1+2_ complex —E_1+2_, E_1+2+9_, E_1+2+9+3_, and E_1+2+9+12_— 12,13,14, we have completed the characterization of the breakpoints of the three inversions leading from the now extinct O_3_ arrangement to the most common arrangements of the telomere proximal part (segment I) of the O chromosome —O_st_^11^, O_3+4_ and O_3+4+8_ (present work)—. Concerning inversions O_4_ and O_8_, our results are consistent with their having both originated (like inversions E_1_, E_9_, E_3_, E_12_ and O_3_) by the staggered double-strand break mechanism. In the case of inversion O_8_, the duplicated fragment that is present in inverted orientation at both breakpoints of the O_3+4+8_ arrangement corresponds to only one breakpoint of the non-inverted O_3+4_ arrangement, similarly to the five previously mentioned inversions of *D. subobscura*. In contrast, duplicates corresponding to both breakpoints of non-inverted O_st_ (or O_3_) chromosomes are present in inverted orientation at each of the two breakpoints of the O_3+4_ arrangement. Even though both fragments present at the proximal breakpoint of the latter arrangement included partial genes, only that corresponding to the distal breakpoint of O_3_ was preserved and could be easily identified as opposed to that corresponding to the O_4_ proximal breakpoint. Indeed, only parts of the duplicated region could be identified through similarity-based searches in the O_4_ proximal breakpoint of the O_3+4_ arrangement. In those cases where the duplicated fragment includes a truncated copy of the gene, the action of purifying selection preserves the functional copy present at the other breakpoint whereas both point and length mutations accumulate through time in the truncated copy due to the relaxation of selection. Also in the case that an inversion originated by ectopic recombination between repetitive elements, the integrity of these elements may be eroded by the accumulation of mutations. Time may thus blur the differential signals left by mechanisms originating inversions.

Concerning the disruptions affecting the breakpoints themselves and also the extended breakpoint regions, our results have revealed (i) one micro-duplication at each the proximal and distal breakpoints of inversion O_8_ in chromosomal arrangement O_3+4+8_, and therefore upon the inversion occurrence; (ii) the independent disruption by paracentric inversions of the short intergenic region between genes *set8* and *Acf* in *D. subobscura* and in the ancestor of the melanogaster subgroup; (iii) the recurrent disruption by paracentric inversions of the short intergenic region upstream of the *TfIIA-S* gene; and (iv) the involvement of genes CG5225 and *Prosβ2R2* in multiple rearrangements in the Drosophila genus that include transpositions, duplications and inversions. It should be noted that genes *set8*, *Acf*, *TfIIA-S* and *Prosβ2R2* share some characteristics concerning their expression, as revealed in *D. melanogaster*. They have at least one Class I insulator near the transcription start site (~50 to 200 nucleotides distance), and *Prosβ2R2* also at the end of the transcription unit. Disruptions at the upstream region of any of these genes as well as on both their upstream and downstream regions —as is the case of the detected interchromosomal transpositions of gene *Prosβ2R2*— would generally not affect their expression given the presence of nearby insulators. There are also diverse indications that the flanking regions of these genes might be breakage-prone. Indeed, the four genes involved in multiple rearrangements are embedded in active chromatin domains with most of them being widely expressed.

Our comparative analysis of gene order changes in *D. subobscura* and across the Drosophila genus has focused on the genes flanking the breakpoints of the former species polymorphic inversions as well as on their neighboring genes. In *D. subobscura*, our molecular characterization of the breakpoints of eight polymorphic inversions (five and three of Muller’s C and E element, respectively) has provided ample evidence for multiple disruptions either of the breakpoints themselves^12^,^14^ or of the extended breakpoint regions^31^. Although our analysis has not revealed any general enrichment in low-complexity repetitive sequences flanking inversion breakpoints with molecular evidence for having been reused, it has revealed the presence of two different snoRNAs generating genes next to a gene involved in two sequential inversions sharing a breakpoint^31^. Our analysis at the long time scale has detected that genes flanking the *D. subobscura* inversion breakpoints and their neighbors have generally been affected by multiple disruptions. It has also identified some genes that have been individually involved in multiple structural rearrangements and more specifically in at least one intrachromosomal or interchromosomal transposition: genes *subito*^12^,^31^, *Lsp1β*^32^ (present work) and *Prosβ2R2* (present work). The expression of these genes would not have been affected by the regulatory elements of their diverse neighboring genes through evolutionary time as supported by the detected nearby insulators. This characteristic might render them elusive to the sieving effects of purifying selection when their flanking regions were disrupted, which would increase their probability of being involved in both polymorphic and fixed rearrangements.

In summary, the characterization (in previous^11^ and present work) of the breakpoints of inversions leading to the most common chromosomal arrangements of the O_3+4_ complex of *D. subobscura* has revealed the presence of inverted duplications only at the inverted arrangements breakpoints, which is only consistent with they having all originated by the staggered-breaks mechanism as also did most inversions of the E_1+2_ complex of this species^12^,^13^,^14^. Moreover, the comparative analysis of the breakpoint regions of inversions involved in the most common arrangements of both complexes across the Drosophila genus further supports that they are prone to participate in evolutionary successful rearrangements as multiple disruptions have been detected at different time scales in these regions. Finally, these analyses have allowed detecting genes involved in multiple and diverse structural rearrangements.

## Materials and Methods

Three homokaryotypic strains of *D. subobscura* were used to molecularly identify the breakpoints of inversions O_4_ and O_8_, and to subsequently sequence their breakpoint regions: strains OF28 (O_st_), *ch cu* (O_3+4_), and OF40 (O_3+4+8_). The OF strains were obtained through over 13 generations of sibmating from isofemale lines established upon collection in Observatori Fabra (Barcelona, Catalonia, Spain), as reported in Puerma *et al*.^12^.

For each inversion, two chromosomal walks were performed to identify its breakpoints using as starting points molecular markers previously mapped in their vicinity. In this procedure, serial sets of probes are *in situ* hybridized on polytene chromosomes to physically map them. This allows advancing (walking) towards each breakpoint until its final identification. The design of probes was based on colinearity blocks between the *D. pseudoobscura* and *D. melanogaster* genomes, as well as on some scaffolds from draft2 of the *D. subobscura* genome sequence (Barcelona Subobscura Initiative [BSI]) as described in Puerma *et al*.^12^. Probes were amplified by PCR using genomic DNA from the *ch cu* strain, biotin labeled and *in situ* hybridized on the corresponding strains.

Oligonucleotides for probes amplification were designed directly on *D. subobscura* sequences. Hybridization signals that were located on the cytological map of *D. subobscura*^28^ allowed walking towards each breakpoint and to eventually cross it. All steps of the *in situ* hybridization procedure were performed as described in Montgomery *et al*.^33^. Digital images at a 400 magnification were obtained using a phase contrast Axioskop 2 Zeiss microscope and a Leica DFC290 camera.

Fragments spanning breakpoints were PCR amplified using TaKaRa DNA polymerase (Takara Bio Inc) in both non-inverted and inverted chromosomes, and oligonucleotides anchored at each breakpoint flanking regions. The amplified fragments were sequenced using primer walking whenever necessary. Amplicons were purified with MultiScreen PCR plates (Millipore) prior to their sequencing with the ABI PRISM version 3.2 cycle sequencing kit. Sequencing products separated on an ABI PRISM 3730 sequencer. All sequences were obtained on both strands and assembled using the DNASTAR package^34^. Sequences newly obtained have been deposited in the EMBL/GenBank Data Libraries under accession numbers LT622817 to LT622824.

### Sequence analysis

All breakpoint regions were annotated with genes through their comparison with the *D. pseudoobscura* genome (FlyBase; http://flybase.org/) using BLAST tools and analyzed with RepeatMasker (http://repeatmasker.org/) to detect transposable elements and other repeated motifs. In order to finely establish each breakpoint and to determine putative duplications resulting from the inversion process, the newly sequenced breakpoint regions of each inversion were compared among them using the Align Sequences Nucleotide BLAST utility at the NCBI webpage.

In order to asses whether the *D. subobscura* breakpoint regions had also been disrupted at the long time scale, we performed a comparative analysis of these regions based on the first 12 sequenced genomes of the Drosophila genus^35^. Our analysis focused on the two genes generally affected by a breakpoint (either because the breakpoint laid in an intergenic region or because the staggered break affected both genes) and in one case on one gene, as it was the only gene affected by the staggered breaks. In order to infer the type of structural change that had led to a particular disruption, we included in the analysis the minimum number of neighboring genes needed for that purpose.

Different FlyBase utilities were used to identify the orthologs of the genes present in the extended breakpoint regions of the *D. subobscura* inversions in Drosophila species other than *D. melanogaster*. Orthologs were initially identified using GBrowse searches. However, the lack of annotation or misannotation of some genes in one or more of the eleven species, as well as a different relative orientation of the genes included in a particular block in some species, led us to manually curate those regions using BLAST tools (including blastn and tblastn).

The phylogenetic analysis of the breakpoint extended regions generally allowed us to infer the type of structural change that had been fixed and in which branch it had originated and become fixed. The disruption of two neighboring genes (or blocks of genes) was considered to be the result of a paracentric inversion when the distance between these genes in the genome with the derived arrangement was very large. Genes within a gene block that in some of the analyzed species are located in a different Muller element than its neighbors are considered to have undergone an interchromosomal transposition. In those cases where the putative transposition affects a single species, the gene location should be considered with some caution as it could also be due to miss-assembly of genome sequences generated by Next Generation Sequencing (NGS).

Class I insulators that act as gene regulatory boundaries allow the independent spatial and temporal expression of adjacent genes. This class of insulators as well as an active chromatin state exhibit a positive association with synteny breaks in the Drosophila genus^36^. Both the function of Class I insulators and the detected associations motivated including these characteristics in our comparative analysis. We therefore retrieved information from FlyBase on Class I insulators and chromatin state at the gene regions included in our analysis.

## Additional Information

**How to cite this article**: Puerma, E. *et al*. Multiple and diverse structural changes affect the breakpoint regions of polymorphic inversions across the Drosophila genus. *Sci. Rep*. **6**, 36248; doi: 10.1038/srep36248 (2016).

**Publisher’s note**: Springer Nature remains neutral with regard to jurisdictional claims in published maps and institutional affiliations.

## Supplementary Material

Supplementary Information
