# Supplementary material for: Multiple and diverse structural changes affect the breakpoint regions of polymorphic inversions across the Drosophila genus
Source: Sci Rep. 2016 Oct 26;6:36248. doi: 10.1038/srep36248 (PMC5080602; doi:10.1038/srep36248)
Supplement: Supplementary Information [file srep36248-s1.pdf]

Supplementary information

Multiple and diverse structural changes affect the breakpoint regions of polymorphic inversions across the Drosophila genus

Eva Puerma, Dorcas J. Orengo, Montserrat Aguadé

Supplementary text: chromosomal walks performed to identify the breakpoints of inversions O_4 and O_8

O_4 inversion breakpoints

The breakpoints of inversion O_4 are cytologically located at sections 94D/94E and 98C/98D on the O_{st} Kunze-Mühl and Müller [28] map and therefore between sections 94E/94D and 98C/98D on the ancestral O_3 arrangement (Figure 1). Markers *AbdA* [11] and *Obp83a/Obp83b* [29] that are located at section 94E and 98D, respectively, were used as starting points to identify the inversion breakpoints in non-inverted chromosomes.

For the proximal breakpoint, we knew in which direction to walk given that this marker had been previously used as starting point to identify the proximal breakpoint of inversion O_3 [11]. Two rather distant probes were initially designed in the colinear fragment between *D. pseudoobscura* and *D. melanogaster* (Supplementary Figure S1). Their mapping by *in situ* hybridization on sections 94E and 94D, respectively, indicated that the breakpoint lies between them. Additional probes were designed in this interval in two rounds, which led us to design a final probe —DO4pOF28— that when *in situ* hybridized on O_{st} chromosomes gave a single strong signal at section 94D/94E, and two strong signals at sections 94E next to 98C and 94D next to 98D when hybridized on $O_{\underline{3+4}}$ chromosomes (Supplementary Figure S2). This result confirmed that the ~5.6-kb long DO4pOF28 probe spanned the proximal breakpoint of inversion O_4 in non-inverted (O_{st} and O_3) chromosomes.

For the distal breakpoint, probes were designed on both sides of the *Obp83a/Obp83b* marker in the *D. pseudoobscura* genome in order to identify in which direction to walk. Although these probes mapped at section 98D, similarly to the *Obp83a/Obp83b* marker, direction could be established from their *in situ* hybridization signals on O_{st} chromosomes. Two additional probes were designed that mapped at sections 98C/D and 98C, respectively, which led us to design a final probe —DO4dOF28— that should include the breakpoint. Its *in situ* hybridization results on O_{st} and $O_{\underline{3+4}}$ chromosomes (Supplementary Figure S2) confirmed that the ~3.8-kb long DO4dOF28 probe spanned the distal breakpoint of inversion O_4 in non-inverted chromosomes as it gave a single strong signal at section 98C/98D on O_{st} chromosomes, and two strong signals at sections 98C next to 94E and 98D next to 94D on $O_{\underline{3+4}}$ chromosomes (Figure 1 and Supplementary Figure S2).

O_8 inversion breakpoints

The breakpoints of inversion O_8 are cytologically located at sections 90D/91A and 94A/94B (Figure 1) according to the Kunze-Mühl and Müller [28] map. Markers previously located near each breakpoint —DP2_4d at section 91A and *trus* at section 93D— were used to initiate the corresponding chromosomal walks. In order to establish in which direction to walk, probes were designed on both sides of each marker based on the *D. pseudoobscura* genome (Supplementary Figure S3).

For the proximal breakpoint, their mapping by *in situ* hybridization did not resolve our question since two of them mapped at section 91A (similarly to the

initial probe) and the third probe did at section 94E. Despite this break of colinearity relative to *D. pseudoobscura*, two new probes were designed on each side. A pair of probes mapped at section 91B (*i.e.,* further away from the breakpoint) whereas one of the other probes pair did again exhibit a colinearity break relative to *D. pseudoobscura*. Despite these results, we could proceed with our walk given the later availability of an improved version of the *D. subobscura* genome (draft2; Barcelona Subobscura Initiative [BSI]) with a scaffold that did not only include the two probes that had mapped nearest to the breakpoint —DO8pGA22022 and DE2_4d— (Supplementary Figure S3), but also other *D. pseudoobscura* orthologous regions not previously explored. New probes could be designed on the newly identified *D. pseudoobscura* regions, with four of them also mapping at section 91A and a fifth one at section 90D (*i.e.,* past the cytological breakpoint). A final set of three probes was designed to identify the breakpoint region. Only probe DO8pC gave a single signal at section 90D/91A on $O_{\underline{3+4}}$ (*ch cu*) chromosomes and two strong signals at the corresponding sections on $O_{\underline{3+4+8}}$ (OF40) chromosomes (Supplementary Figure S4). This result confirmed that the ~4.8-kb long DO8pC probe spanned the proximal breakpoint of inversion O_8 in non-inverted ($O_{\underline{3+4}}$) chromosomes.

For the distal breakpoint, results of the *in situ* hybridization of the first round of probes allowed establishing the direction in which to walk since probes on the distal side of *trus* mapped at either section 93C/D or 94A (*i.e.*, moving closer to the breakpoint; Supplementary Figure S3). A set of three new probes was designed, with the first two also mapping at section 94A and the third one at section 85A/B. Despite this colinearity break, we could proceed with our walk

by identifying, through draft2 of the *D. subobscura* genome (BSI), a new *D. pseudoobscura* region to explore (Supplementary Figure S3). A third set of probes was designed that mapped at sections 94A, 94B, 94B/C and 94C in $O_{\underline{3+4}}$ chromosomes (Supplementary Figure S3). According to the Kunze-Mühl and Müller [28] map, the first two probes should delimit the breakpoint. Results of the *in situ* hybridization of the five probes on $O_{\underline{3+4+8}}$ (OF40) chromosomes revealed, however, that the third and fourth probes were those that delimited the breakpoint (Supplementary Figure S3). An ~7.2-kb long final probe —DO8dD— was designed that gave a single strong signal on $O_{\underline{3+4}}$ chromosomes, and two strong signals on $O_{\underline{3+4+8}}$ chromosomes. These results indicate that the distal breakpoint of inversion O_8 is at section 94B/C of the Kunze-Mühl and Müller [28] map.

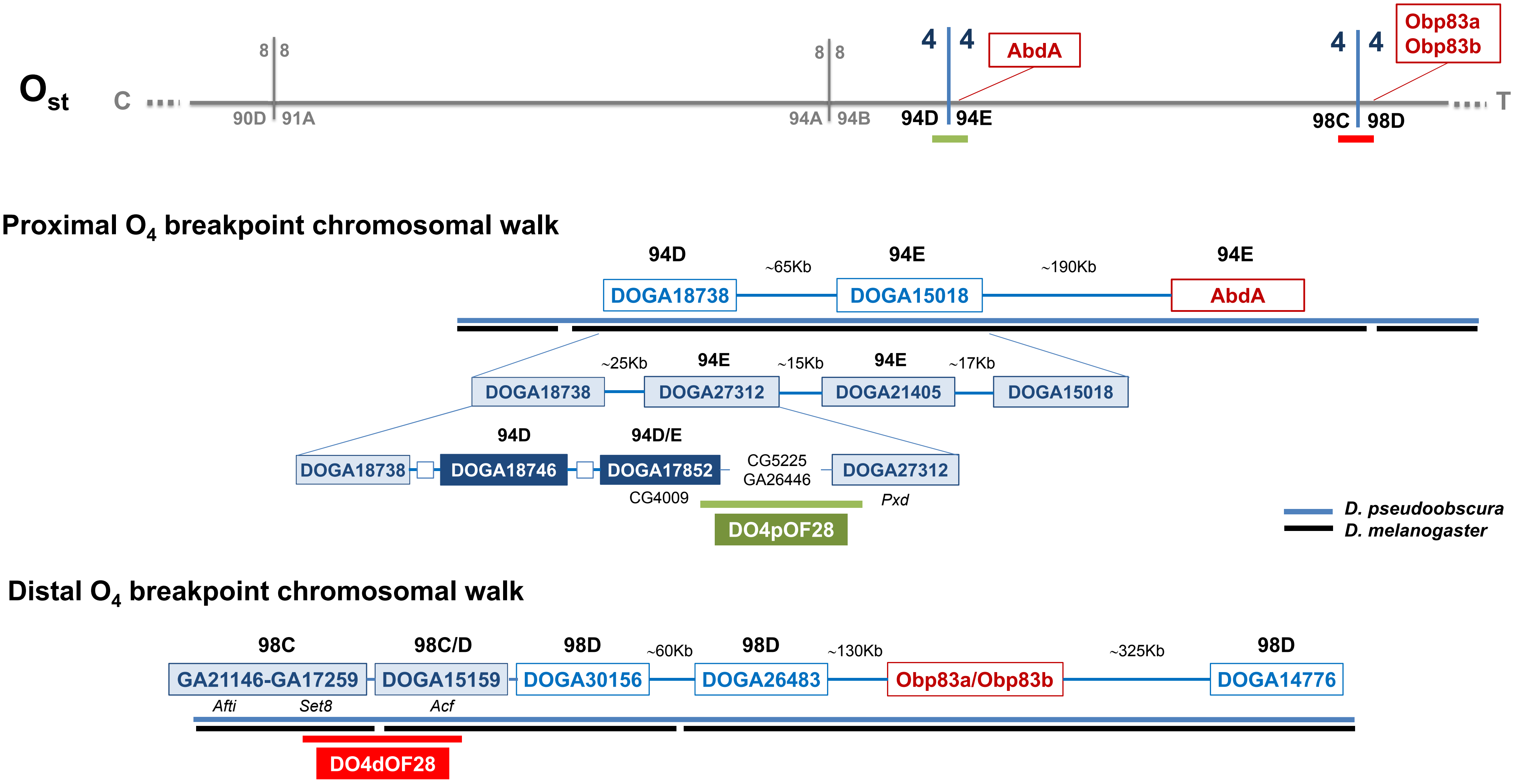

Supplementary Figure S1. Inversion O_4 breakpoints chromosomal walks. Schematic representation of the breakpoints of inversion O_4 on O_{st} chromosomes (upper part) and chromosomal walks performed to identify the inversion breakpoint regions in O_{st} (strain OF28) chromosomes (lower part; not at scale). The blue horizontal lines represent regions of the *D. pseudoobscura* genome, whereas black horizontal lines represent colinear blocks in *D. melanogaster* relative to the former species. Breakpoint regions and probes spanning the breakpoints are color-coded as in Figure 1. In chromosomal walks, probe names and their location (section) on the Kunze-Mühl and Müller (1958) map of *D. subobscura* are indicated above those lines, with probes used to initiate walks highlighted in dark red. When three or more coding regions separate two probes in *D. pseudoobscura*, their approximate distance is given, with a small box indicating one to three genes. C, centromere. T, telomere.

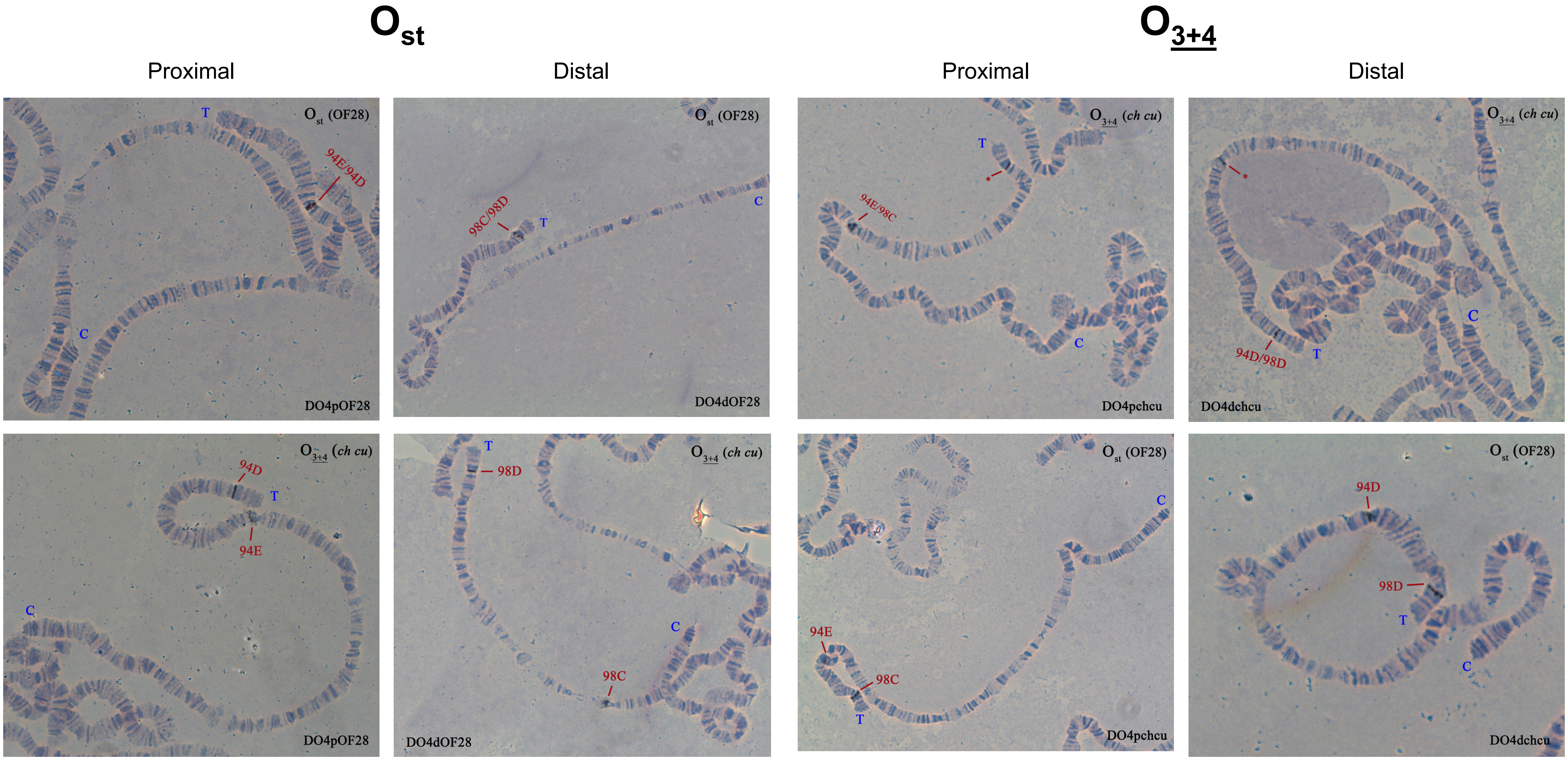

Supplementary Figure S2. Inversion O_4 *in situ* hybridization results. Results of the *in situ* hybridizations performed on chromosomal arrangements O_{st} (OF28) and $O_{\underline{3+4}}$ (*ch cu*) using the probes spanning the breakpoints in either O_{st} chromosomes (left side), or $O_{\underline{3+4}}$ chromosomes (right side). Hybridization signals are marked with a red line and their cytological location is also indicated in red. Note that probes spanning the $O_{\underline{3+4}}$ breakpoints give a secondary signal marked with a red asterisk when hybridized on $O_{\underline{3+4}}$ (*ch cu*) chromosomes (see main text). Probes are named as in Figure 1.

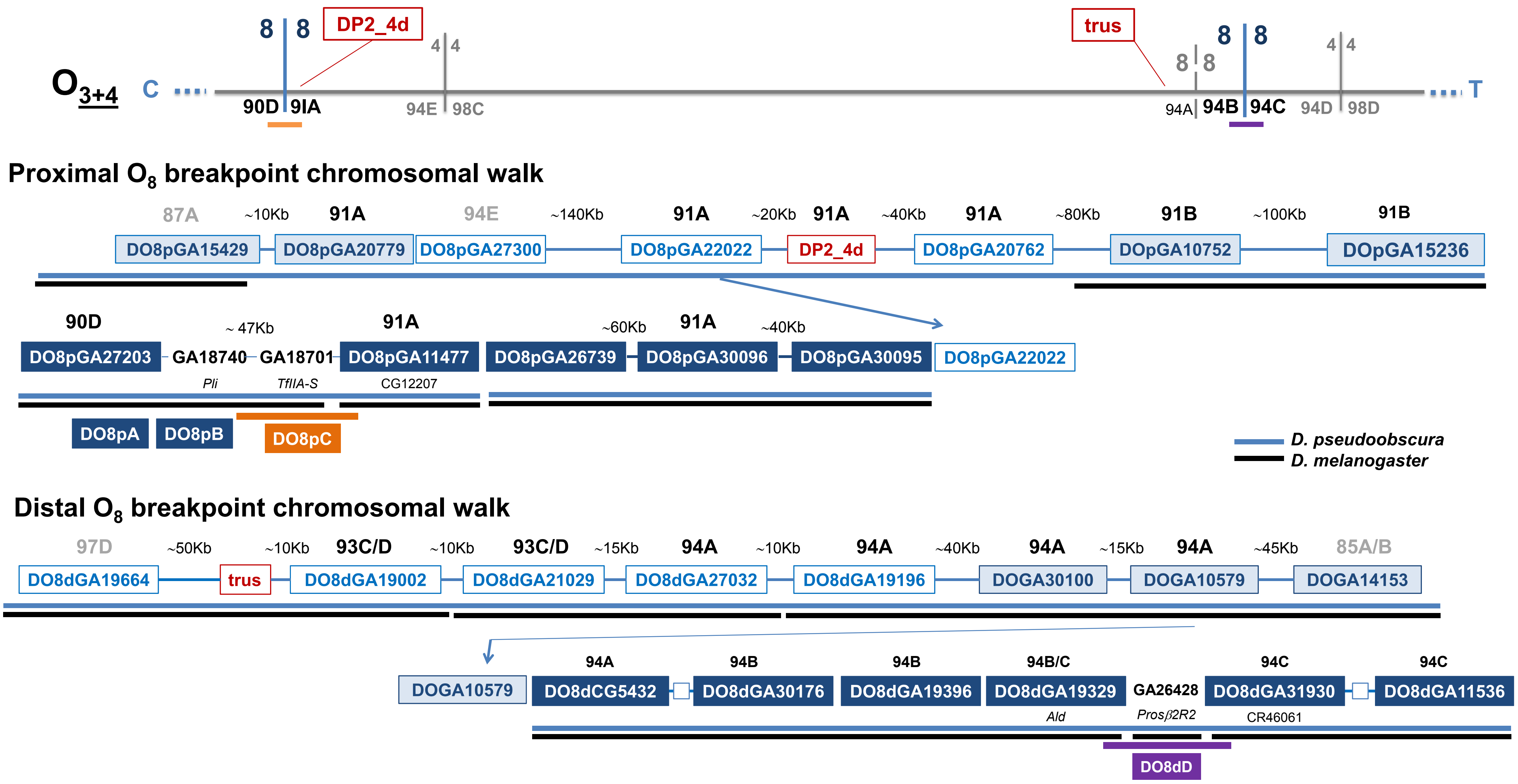

Supplementary Figure S3. Inversion O_8 breakpoints chromosomal walks. Schematic representation of the breakpoints of inversion O_8 on $O_{\underline{3+4}}$ chromosomes (upper part) and chromosomal walks performed to identify the O_8 inversion breakpoints in $O_{\underline{3+4}}$ (*ch cu*) chromosomes (not at scale). The blue horizontal lines represent regions of the *D. pseudoobscura* genome. Black horizontal lines represent colinear blocks in *D. melanogaster* relative to the former species. Breakpoint regions and probes spanning the breakpoints are color-coded as in Figure 1. In chromosomal walks, probe names and their location (section) on the Kunze-Mühl and Müller (1958) map of *D. subobscura* are indicated above those lines, with probes used to initiate walks highlighted in dark red. When three or more coding regions separate two probes in *D. pseudoobscura*, their approximate distance is given, with a small box indicating one to three genes. C, centromere. T, telomere.

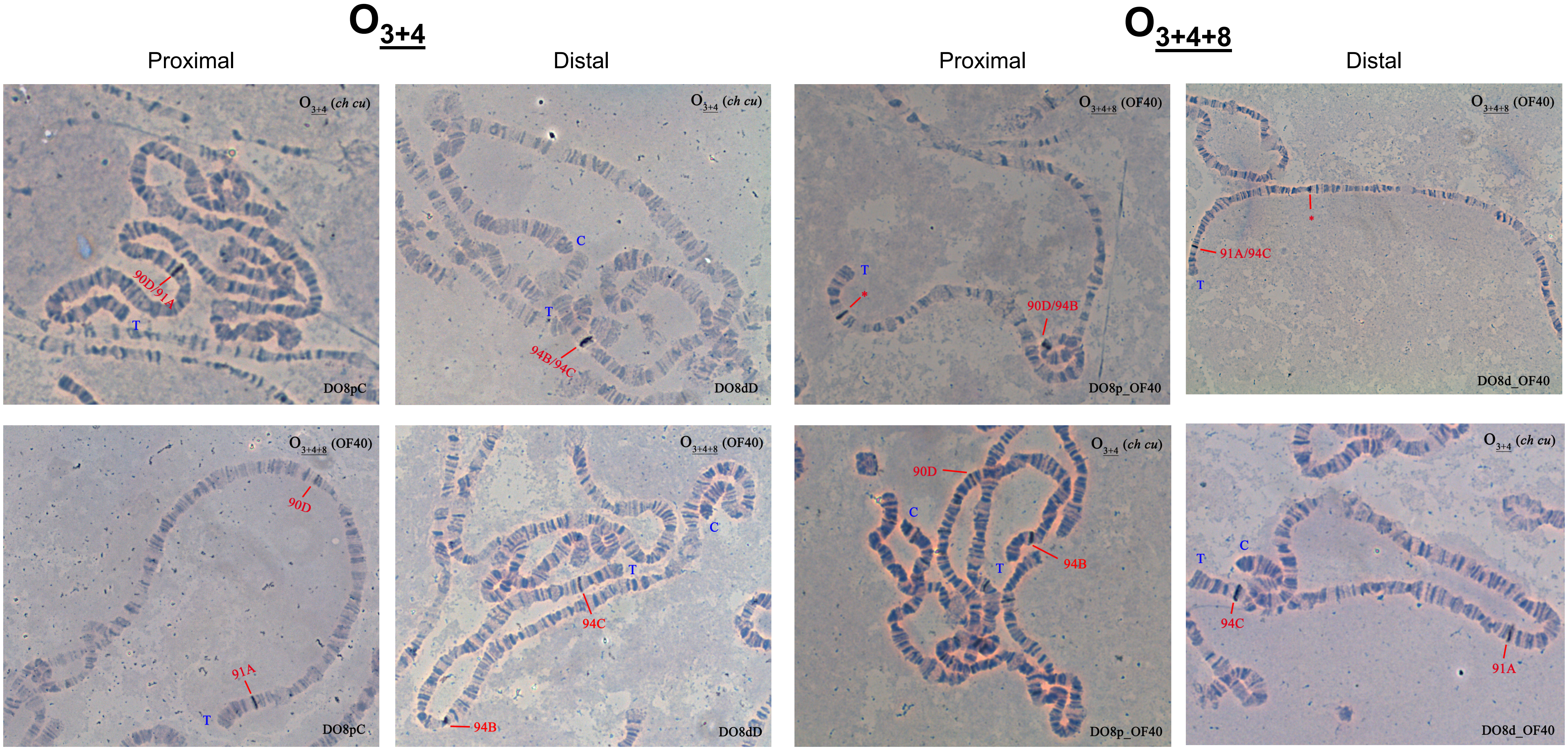

Supplementary Figure S4. Inversion O_8 *in situ* hybridization results. Results of the *in situ* hybridizations performed on chromosomal arrangements $O_{\underline{3+4}}$ (*ch cu*) and $O_{\underline{3+4+8}}$ (OF40) using probes spanning the breakpoints in either $O_{\underline{3+4}}$ chromosomes (left side), or $O_{\underline{3+4+8}}$ chromosomes (right side). Hybridization signals are marked with a red line and their cytological location is also indicated in red. Note that probes spanning the $O_{\underline{3+4+8}}$ breakpoints give a secondary signal marked with a red line and asterisk when hybridized on $O_{\underline{3+4+8}}$ (OF40) chromosomes (see main text). Probes are named as in Figure 1.

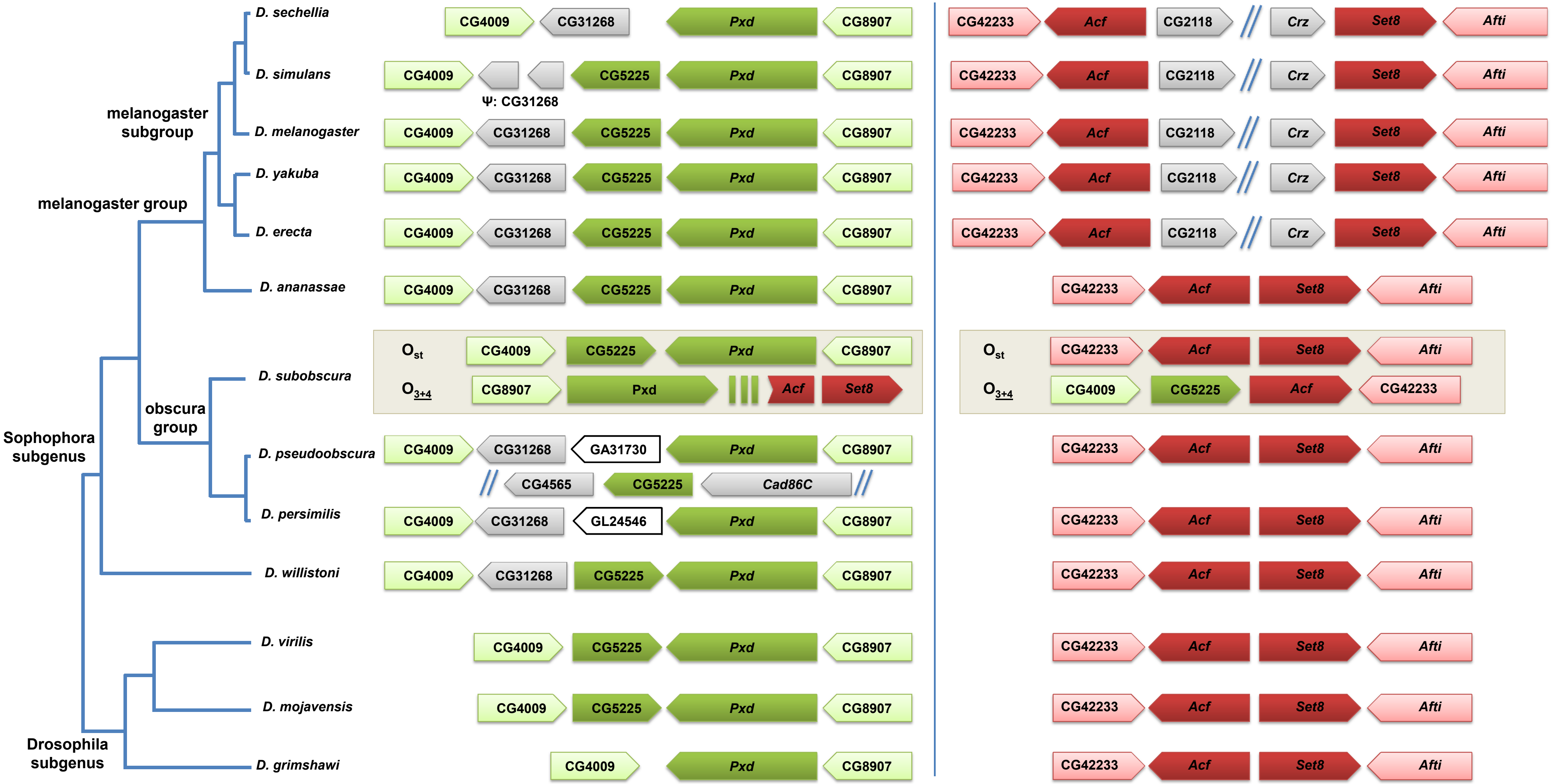

Supplementary Figure S5. Schematic representation of the genes flanking inversion O_4 breakpoint across the Drosophila phylogeny. Genes adjacent to the proximal breakpoint and their neighboring genes in *D. subobscura* are represented by dark and light green boxes, respectively, whereas those flanking the distal breakpoint in *D. subobscura* are represented by dark and light red boxes, respectively. Grey or clear boxes represent all other genes (with orthologs and orphan, respectively). All genes are located in Muller's E element and they are named according to their *D. melanogaster* orthologs. In *D. subobscura*, the location of genes in both the O_{st} and $O_{\underline{3+4}}$ arrangements is given. Gene blocks are ordered as in the O_{st} chromosomal arrangement of *D. subobscura.* Inclined parallel lines indicate a very large intervening fragment.

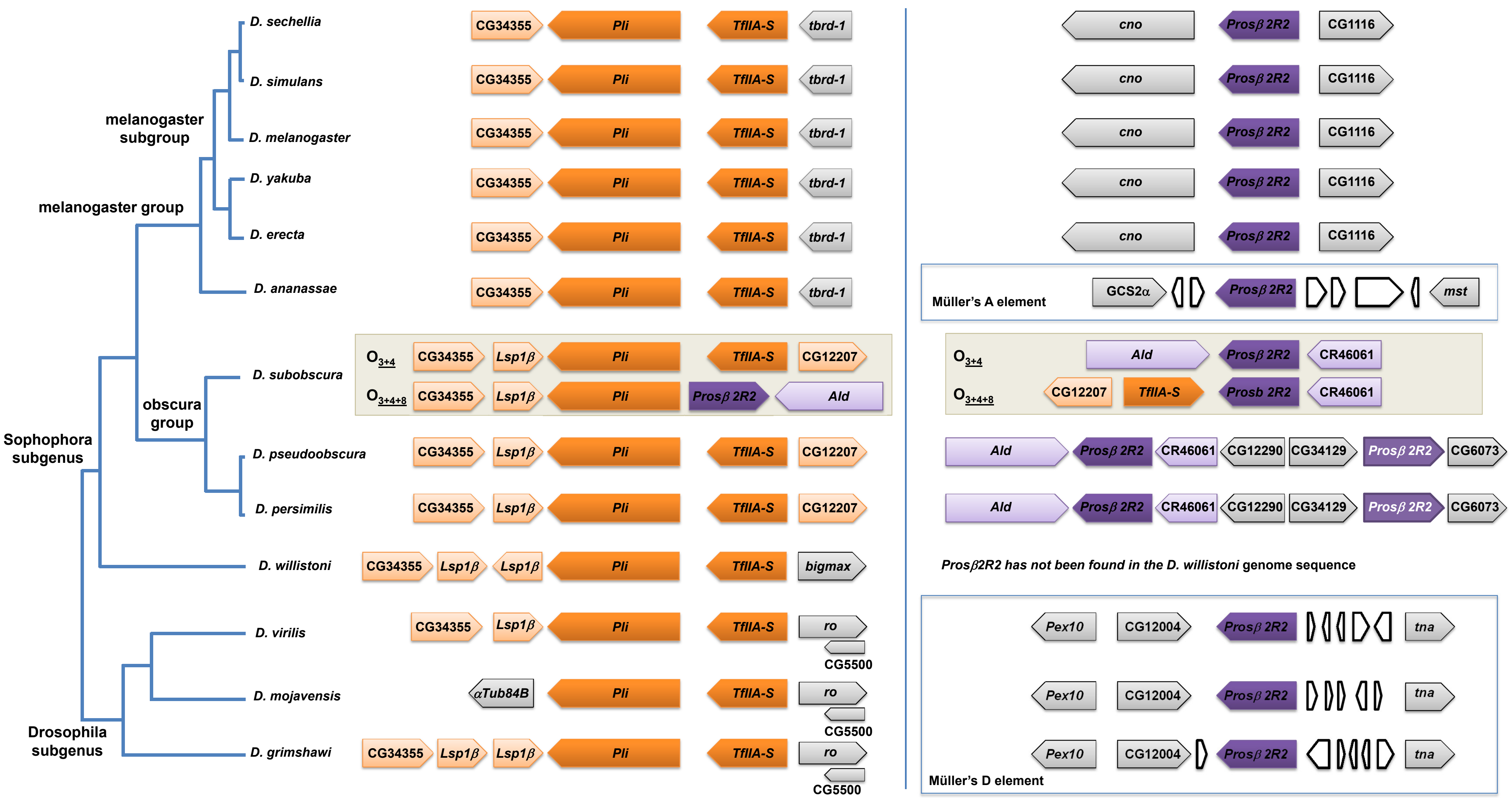

Supplementary Figure S6. Schematic representation of the genes flanking inversion O_8 breakpoint across the Drosophila phylogeny. Genes adjacent to the proximal breakpoint and their neighboring genes in *D. subobscura* are represented by dark and light orange boxes, respectively, whereas those flanking the distal breakpoint in *D. subobscura* are represented by dark and light purple boxes, respectively. Grey and clear boxes represent all other genes (with orthologs and orphan, respectively). Genes are located in Muller's E element except gene *Prosβ2R2* in the distal breakpoint that is located in Muller's A element in *D. ananassae* and in Muller's D element in species of the Drosophila subgenus. Genes are named according to their *D. melanogaster* orthologs. In *D. subobscura*, the location of genes in both the $O_{\underline{3+4}}$ and $O_{\underline{3+4+8}}$ arrangements is given. Gene blocks are ordered as in the O_{st} chromosomal arrangement of *D. subobscura.*
